# Comparison of implant stability after different implant surface treatments in dog bone

**DOI:** 10.1590/S1678-77572010000400016

**Published:** 2010

**Authors:** Sun-Jong KIM, Myung-Rae KIM, Jae-Suk RIM, Sung-Min CHUNG, Sang-Wan SHIN

**Affiliations:** 1 DDS, MSD, PhD, Oral and Maxillofacial Surgery, School of Medicine, Ewha Womans University, Seoul, Korea.; 2 DDS, MSD, PhD, Oral and Maxillofacial Surgery, School of Medicine, Ewha Womans University, Seoul, Korea.; 3 DDS, MSD, PhD, Oral and Maxillofacial Surgery, College of Medicine, Korea University, Seoul, Korea.; 4 DDS, MSD, PhD, Well Dental Clinic, Seoul, Korea.; 5 DDS, MPH, PhD, Advanced Prosthodontics, Institute for Clinical Dental Research, Graduate School of Clinical Dentistry, Korea University , Seoul, Korea.

**Keywords:** Implant surface, Primary stability, Bone quality, Histomorphometry, Resonance frequency analysis

## Abstract

**Objectives:**

The purpose of this investigation was to evaluate the effects of different implant surface treatments on implant stability in dog mandibles.

**Material and Methods:**

A total of 30 implants (Dentium Co, Seoul, Korea) were placed in 5 dog mandibles. Bone quality was assessed at each site. Implant stability was evaluated using 2 different methods. An Osstell™ resonance frequency analyzer (RFA) was used to determine the stability at baseline (day 1), and 3, 6 and 10 weeks after surgery. Animals were euthanized 10 weeks after implant installation. Specimens were obtained and submitted to the laboratory processing. Sections were stained with hematoxylin and eosin for histologic and histomorphometric analyses. All implantation sites in dog mandibles demonstrated bone types II and III.

**Results and Conclusions:**

All implants showed good primary stability at baseline in terms of insertion torque. The results of this study suggest that surface treatment may have significant effects on biological stability 3 weeks after implant placement. Further studies are needed to confirm these initial observations in poor quality bone.

## INTRODUCTION

Implant stability is one of the crucial factors for a long-term success of osseointegration. There are different methods of measuring implant stability, such as percussion, radiograph, Periotest^®^ (Siemens AG, Modautal, Germany), Dental Fine Tester^®^ (Kyocera, Kyoto, Japan), thread cutting force and the reverse torque test. However, they have been criticized for lack of resolution, poor sensitivity and susceptibility to being influenced by the operator. Resonance frequency analysis (RFA) offers a clinical, non-invasive measure of stability and presumed osseointegration of implants^12-13,18^.

Implant primary stability can be obtained by choosing an implant that matches bone quality and by applying an appropriate surgical technique according to the bone quality^3^. Sennerby, Thomsen and ericson^20^ (1992) analyzed the healing process in the early stage of implantation by performing a research on reaction of bone tissue in rabbit cortical and cancellous bone. In this study, those authors emphasized the importance of cortical bone fixation. Although it is relatively easy to obtain implant primary stability in cortical bone, it is somewhat difficult to achieve implant primary stability in areas such as the maxillary molar area where severe bone resorption, poor bone quality and lack of bone quantity are present. Some implant researchers who have been interested in soft bone have attempted to overcome this limitation by implant design and surface treatment. Glauser, et al.^5^ (2001) have reported that implant design and surface treatment have a significant influence on soft bone.

The aim of this study was to evaluate the effects of surface treatment on implant primary stability using RFA and histomorphometric analysis.

## MATERIAL AND METHODS

**Figure 1:**
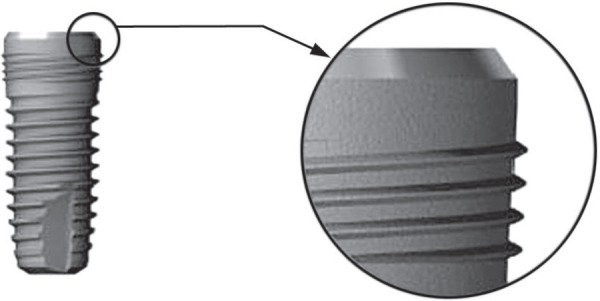
Implants used in this study (3.4 mm in diameter, 6 mm in length)

Five male mongrel dogs weighing over 10 kg were used in this study. Animal selection/ management and surgery protocol followed the routines approved by the Animal Care and Use Committee, Korea University, Seoul, Korea. Animals had access to a standard laboratory diet and water until the beginning of the study. A total of 30 cylindrical implants (Implantium^®^, Dentium Co., Seoul, Korea, 3.4 mm x 6 mm) were used in this study (Figure 1).

Implants were divided into 3 groups: those which were machined and did not undergo any surface treatment (group 1), those which were treated by SLA (group 2) and those which were anodized by oxidized electricity using pulse power (Autoelectric Co., Seoul, Korea) (group 3). The surface roughness of all groups was measured at the top of the thread using a confocal laser scanning profilometer (TopScan3D^®^; UBM Messtechnik GmbH, Germany) (Table 1).

**Tabela 1:** Implants used in this study (3 groups)

**Group**	**Surface**	**Material and surface characteristics**	**Roughness (Sa)**	**Number**
				
1	Machined	Smooth titanium,	0.86 |jm	10
		as-machined		
2	SLA	Sand blasted with large grit acid etched	1.76 jm	10
				
3	Anodized	Anodic oxidation	1.02 jm	10
Total				30

**Figure 2:**
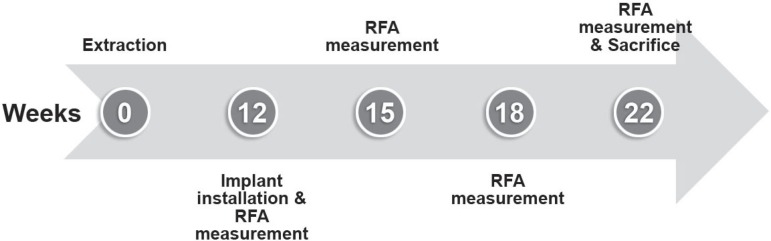
Experimental time points of this study

**Figure 3:**
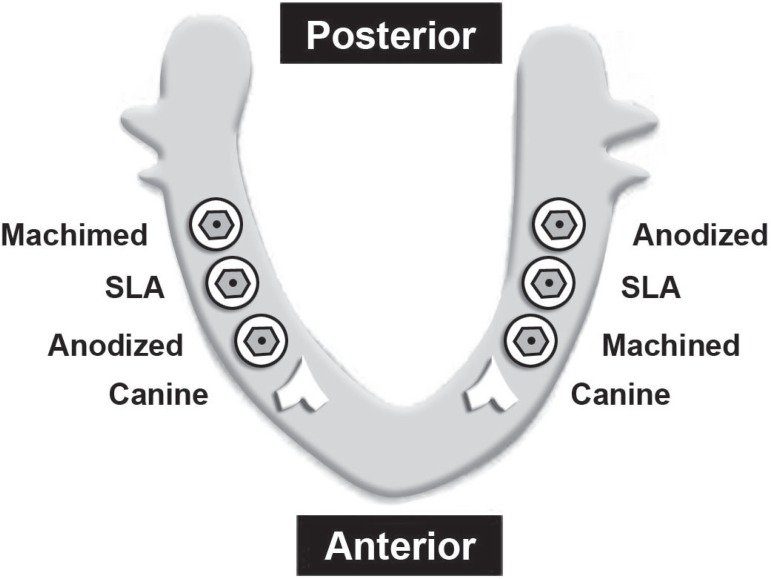
Implant position in the dog’s lower jaw

Animals were preanesthetized by subcutaneous injection of buprenorphine HCl (Hanlim Co., Seoul, Korea, 0.02 mg/kg)/acepromazine (0.1 mg/kg)/ atropine (0.02 mg/kg). They were then sedated with methohexital (5 mg/kg) and maintained on gas anesthesia (2% isoflurane/O_2_). After scrubbing the surgical site with potadine, 1 mL of 2% lidocaine (Yu-Han Co., Seoul, Korea; 1:100,000) was injected into each surgical site for local anesthesia. In order to create an edentulous alveolar ridge, 4 mandibular premolar teeth were extracted from both sides of the lower jaw. After 3 months of healing period, implants were installed under general anesthesia. This experiment was undertaken as follows (Figure 2). For each implant, the insertion torque (IT), which represents the cutting resistance of bone when its rotation is stopped, was registered in Ncm (INTRAsurg 300, KaVo, Bieberach, Germany). The implants were placed at the bone level. After stable installation of implants, cover screws were connected to them (Figure 3)

All implantation sites tested in dog mandibles were demonstrated to be bone type II or III. The surgical wound was sutured with 3-0 nonabsorbable sutures (Mersilk^®^, ethicon Co., U.K.). In order to prevent infections, kanamycin (50 mg/kg, Dong-A Co. Seoul, Korea) was injected intramuscularly for 7 days after surgery. One week after the operation, the sutures were removed, and a soft diet was provided for 2 weeks after surgery. A regular chow diet was supplied after 2 weeks. Implant Stability Quotient (ISQ) was recorded using an Osstell mentor^TM^ (Integration Diagnostics AB, Gothenburg, Sweden) at baseline (the day of surgery), and 3, 6 and 10 weeks after implant installation. All items measured in the experiment were recorded with an Osstell mentor Data Manager (OmDM). The program also calculated the mean and standard deviations. Generalized Linear Model in Statistical Package for the Social Science (SPSS) for Windows (ver. 11.0, SPSS Inc.) was used for comparative analysis among the groups.

Animals were sacrificed 10 weeks after implant installation, and histomorphometric analysis was performed in order to measure the degree of osseointegration. Specimens including implant (N=30) were prepared for histomorphometric analysis. The specimens were stained with hematoxylin and eosin for light microscopy. Histomorphometric analysis was performed by attaching a Kappa Dx30 digital camera (Optoelectronics, Gleichen, Germany) on the light microscope (Olympus BX51, Olympus Co., Tokyo, Japan) and transferring digital images to a computer monitor. Then, a quantitative analysis was performed using Kappa image base metro (Kappa Opto-electronics, Germany) as an image-analysis software: A. The bone-to-implant direct contact ratio (BIC) was measured at the thread using a x40 magnification; B. The mineralized bone ratio was calculated by measuring the total surface area of all threads (bone density: trabeculae, bone volume/ total volume).

## RESULTS

**Figure 4:**
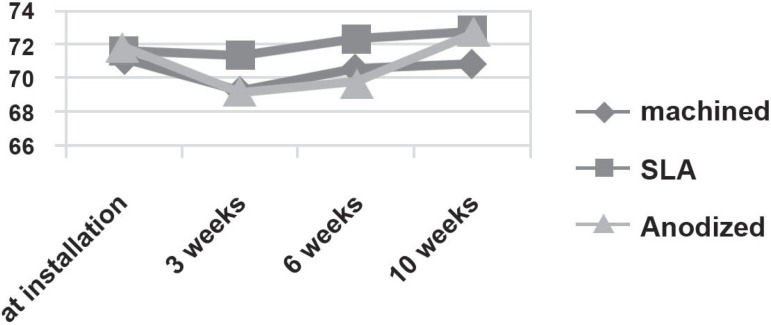
Changing pattern of implant stability quotient during the healing period following implant installation in different types of implants (P<0.05)

**Figure 5:**
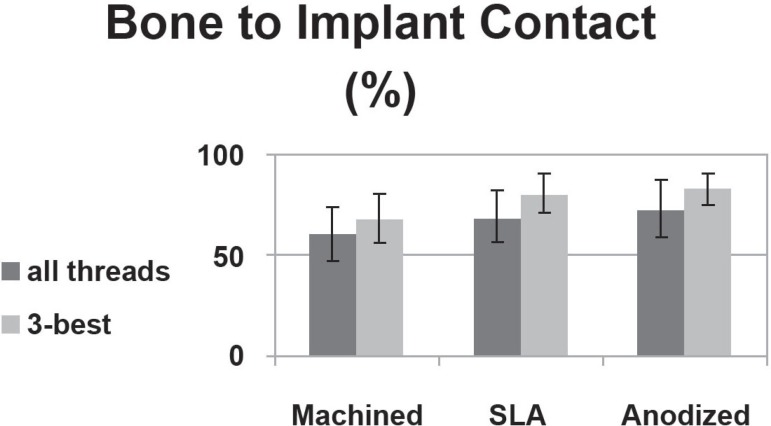
The bone-to-implant contact (BIC) ratio in different types of implant

**Figure 6:**
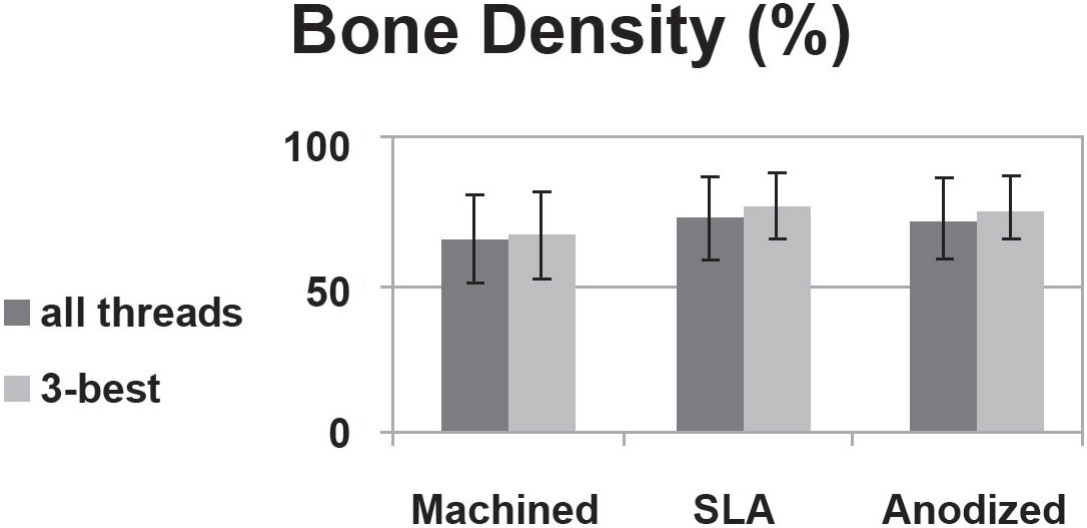
Bone density in different types of implants

All implants showed a mean insertion torque value of 18.12±6.53 Ncm as compared to a set torque of 40 Ncm and higher initial stability at baseline with an ISQ value than recommended ISQ (Implant Stability Quetient) value 70. No statistically significant differences were found among the 3 groups immediately after implantation (P>0.05). The 3 groups showed similar ISQ changing patterns 0, 3, 6 and 10 weeks after implant installation, with a decreasing pattern during the first 3 weeks and increasing 3 weeks later (P<0.05) (Table 2) (Figure 4). The bone-to-implant contact ratios of groups 1, 2 and 3 in all threads were 60.8%, 69.6% and 73.6%, respectively. There was a significant difference in the contact ratio between groups 1 and 3 (P<0.05). The bone-to-implant contact ratio of groups 1, 2 and 3 in the 3 best continuous threads with abundant bone quantity showed values of 68.6%, 81.2% and 83.2%, respectively. There was a significant difference in the contact ratio between groups 1 and 2 and between groups 1 and 3 (P<0.05) (Figure 5). For all threads, bone density was 65.4% in group 1, 72.5% in group 2 and 71.1% in group 3. There was no significant difference in bone density between the 3 groups (P>0.05). For the 3 best continuous threads with abundant bone quantity, the bone density was 66.3% in group 1, 76.5% in group 2 and 75.7% in group 3 (Figure 6).

**Tabela 2:** ISQ during healing period

**Surface**	**at installation**	**3^rd^week**	**6^th^week**	**10^th^week**
				
Machined	71.33±2.42	69.33±3.14	70.67±2.58	70.83±3.31
SLA	71.67±3.33	71.36±3.72	72.33±1.63	72.83±1.94
Anodized	71.83±2.48	69.17±5.91	69.83±5.04	72.67±1.75

## DISCUSSION

Dental implant surface has shown rapid evolution during the past few years^6,17,23^. This is because implant surface can have a significant effect on long-term implant maintenance. Direct bone contact with metallic substrate can be achieved in thin-film coated implants^16^.

It has been demonstrated that implant stability depends on the contact patterns between bone and implant^7^. Stable initial fixation may have an overt effect on long-term implant stability^3^. Several^1,21,22^ reports indicate cortical bone, not bone in general to be mainly responsible for implant stability.

Meredith, et al.^12,13^ (1996, 1997) developed a reversible experimental method to measure implant stability quantitatively. This reversible method allowed the measurement of osseointegration depth according to elapsed time^15^. Friberg, et al.^3^ (1999) have indicated that in compact bone, stability decreases as time passes despite excellent bone adhesion and increased contact between the implant and bone, whereas in soft bone, stability increases as time passes. Those authors have also proposed that the initial implant stability decreases as a result of bone compression caused by mechanical bone relaxation, biological changes during the primary bone recovery stage, and initiation of marginal bone resorption. Concerning the changes in stability according to elapsed time, Glauser, Portmann and Ruhstaller^5^ (2001) installed 20 TiUnite and 27 machined implants in the molar area of 9 and 15 patients, respectively. They performed RFA for 6 months after implantation, which showed a decreasing pattern for the first 3 weeks and then an increasing pattern thereafter.

In this study, ISQ values at baseline, 3, 6 and 10 weeks after implant installation were significantly different among 3 groups. There was a changing pattern of ISQ values that slightly decreased from implantation to 3 weeks post-implantation and increased thereafter in all groups.

SLA treatment, a combination of blasting and acid treatment, is performed by sand blasting the implant with 25-50 μm Al_2_O_3_ particles and then etching with HCl/H_2_SO_4_ mixed solution. Cochran et al.^2^ observed an increase in alkaline phosphatase activity, DNA absorption in 3H chimicin's and collagenase by biochemically testing the condition of cultured cells in the SLA-treated titanium phase.

When the anodizing method is used, a thick porous oxide film is formed. This film can increase frictional force between implant and bone, and bone quantity and bone quality changes depending on the pore size^10^. Also, an improved surface accelerates recovery in the early stage by protein absorption, platelet accumulation and activity, fibrin maintenance, and augmentation of the surrounding bone tissues^9^. When bone quality is poor, the contact rate between bone and implant decreases up to below 25%, hindering implant primary stability which is an important factor in successful osseointegration. In this study, the 3 groups did not show any significant difference in primary stability because the experimental dogs had type II and III bone quality mandibles and because the implants were completely fixated in all groups. This is in agreement with the findings of O'Sullivan, et al.^14^ (2004), who reported that implant primary stability did not show any significant difference between type II and IV bone. They also found a significant difference in the mean maximum insertion torque between type II and IV bone and between type III and IV bone, whereas no significant difference was noted between types II and III bone. Several reports have demonstrated the relationship between surface treatment and bone quality. The authors have suggested that factors related to bone density and implant diameter/length may affect the level of implant primary stability. Furthermore, greater stability was observed in male patients than in female patients. High implant primary stability was achieved in all jaw regions, although the use of thinner drills and/or tapered implants cannot fully compensate for the effect of soft bone^16^. In contrast, Ganeles, et al.^4^ (2008) reported that even in poor bone quality, SLActive surface were safe and predictable when used in immediate and early loading procedures. The survival rate was comparable with that of conventional loading. The mean bone-level change was not deemed to be clinically significant and corresponded well with typical bone resorption observed in conventional implant loading. Lazzara, et al.^11^ (1999) reported that the osseointegration rate of a dual-acid-etched implant was twice as high as that of a machined implant (73% versus 34%). In this study, the rate was higher in implants with anodized surfaces, followed by those with SLA surfaces and those with machined surfaces. In other words, surfacetreated implants showed a higher osseointegration rate (69%-76%) than machined implants (60%) (P<0.05). Johansson, et al.^8^ (1998) installed pure titanium and titanium-aluminum-vanadium in rabbit bone and measured bone density 1, 6 and 12 months after implantation. Bone density did not show significant differences around different implant surfaces. In the present study, bone density was higher in SLA-treated implants, followed by anodized and machined implants, but the differences were not statistically significant (P>0.05). In SLA-treated implants, there was a significant difference in bone density between cancellous and compact bone. This result suggests that there is a significant difference in the bone response according to bone quality in textured surface. It is well known that implants with rough surfaces increase the contact surface area between implant and bone and thus improve the success rate of implants. Implants with rough surfaces show a high success rate and an excellent clinical outcome when used in poor quality bone^20^. Wennerberg, et al.^24^ (1993) stated that the bone-to-implant contact ratio was higher in titanium implants with surface roughness (Sa) of about 1.4 μm than in smoother implants (Sa=0.7-1.2 μm) or rougher implants (2.2 μm). In the present study, the bone-to-implant contact ratio was higher in anodized surfaces with a roughness of 1.02 μm (73.6%±14.4%) and SLA-treated surfaces with a roughness of 1.76 μm (69.6%±12.5%) than in machined surfaces with a roughness of 0.86 μm (60.82%±13.11%).

In the present study, a good condition was created for implant primary stability by providing microthreads and connecting them to a compact bone area. Marginal bone resorption depending on elapsed time was minimized. It is considered that there were no significant differences among the 3 groups because of the good mechanical stability and the microthread design, which reduced marginal bone resorption.

## CONCLUSION

The surface treatment had insignificant effects and did not affect implant stability in a compact bone (dog mandible). Further studies are needed to confirm the effects of microthreads on implant stability in bone.
